# Oral delivery of water-soluble compounds to the phytoseiid mite *Neoseiulus californicus* (Acari: Phytoseiidae)

**DOI:** 10.1371/journal.pone.0223929

**Published:** 2019-10-16

**Authors:** Noureldin A. Ghazy, Takeshi Suzuki

**Affiliations:** 1 Graduate School of Bio-Applications and Systems Engineering, Tokyo University of Agriculture and Technology, Koganei, Tokyo, Japan; 2 Agriculture Zoology Department, Faculty of Agriculture, Mansoura University, El-Mansoura, Egypt; 3 Japan Society for the Promotion of Science, Chiyoda, Tokyo, Japan; Zhejiang University, CHINA

## Abstract

Phytoseiids are predatory mites that prey on other mites and small arthropods, and several species are used in commercial agriculture for biological control of pests. To optimize phytoseiid mites’ use in biocontrol, an efficient method for oral delivery of test compounds is required to assess their sensitivities to pesticides, RNAi for gene functional analysis and artificial diets. Here we developed four methods for oral delivery of a solution of xenobiotics to different life stages of the commercially available generalist predatory mite *Neoseiulus californicus*: (i) soaking mites in the solution, or allowing them to feed on (ii) spider mites soaked in the solution, (iii) a solution droplet, or (iv) solution-saturated filter paper. As measured by ingestion of a tracer dye, the droplet-based feeding system was most efficient; the dye was observed in the alimentary canal of >90% test mites of all life stages, with no mortality. The droplet-based feeding system was also effective for the commercially available specialist predatory mite *Phytoseiulus persimilis*, with >80% delivery efficiency. This study paves the way for development of methods for high-throughput RNAi and for toxicological or nutritional assays in phytoseiid mites.

## Introduction

Predatory phytoseiid mites (Acari: Phytoseiidae) are an economically important group of mites that prey on other mites and small arthropods. Several phytoseiid mites are widely and extensively used for agricultural biocontrol of pest mites and several insect species, as an alternative to chemical pesticides [1]. For example, *Neoseiulus californicus* (McGregor) and *Phytoseiulus persimilis* Athias-Henriot, as well as several other species, are currently available as commercial products for use in managing spider mite populations in greenhouses and open fields [2–5].

Delivery of xenobiotics to predatory mites is becoming an important research target because it can aid in evaluating selective pesticides that do not affect beneficial mites and in testing of new and promising genetic-based pest management technologies such as RNA interference (RNAi)–mediated gene silencing. Recent progress in RNAi technologies paves the way for sophisticated functional genomic studies of many organisms and has the potential to be used as a novel measure for pest control [6–8]. Although RNAi is specific to the target gene sequence, its application for pest control requires preliminary investigation to avoid off-target effects on non-target organisms.

To date, few methods have been developed for oral delivery of test compounds to phytoseiid mites. As part of a functional genomic study, double-stranded RNA (dsRNA) that triggers RNAi was delivered to the phytoseiid mite *Metaseiulus occidentalis* by allowing mites to feed on a solution droplet (10 μL) containing dsRNA on a Parafilm membrane [9]. However, the method has some drawbacks, such as evaporative loss of the dsRNA solution and mite escape from the membrane. Ozawa et al. (2012) allowed *P*. *persimilis* to feed on a relatively large volume (500 μL) of dsRNA solution delivered via a saturated piece of cotton (≈1 cm diameter) [10]. Ideally, an effective method for oral delivery of test compounds to mites should minimize mite escape, evaporative loss, and the volume of test solution required.

The predatory mite *N*. *californicus* and *P*. *persimilis* have two distinct lifestyles and feeding habits; the former is a generalist and the latter is an extreme specialist [2, 11]. *Neoseiulus californicus* consumes a wide range of prey, including, in addition to spider mites, other pest mite species, small insects, and pollen [12]. *Phytoseiulus persimilis* is a specialist predator of spider mites of the genus *Tetranychus* [13, 14]. Therefore, developing a protocol that facilitates the efficient delivery of a test solution to these two distinct species could aid in studying various aspects of mite biology, such as liquid-based artificial diets and prey–predator interactions.

In this study, we tested four methods for oral delivery of small molecules in solutions to the generalist mite *N*. *californicus*: (i) soaking in the solution, or feeding on (ii) spider mites soaked in the solution, (iii) a solution droplet, or (iv) a solution-saturated filter paper. We also evaluated solution droplet as the most efficient delivery method for use with the specialist *P*. *persimilis* to determine its wider applicability among other phytoseiid mites.

## Materials and methods

### Mites

Commercial populations of *N*. *californicus* and *P*. *persimilis* were obtained from Sumika Technoservice (Takarazuka, Japan) and Koppert Biological Systems (Berkel en Rodenrijs, The Netherlands), respectively, in 2017. Both populations were maintained in the laboratory (25°C, 50% RH, LD 16:8) on detached kidney bean leaves (*Phaseolus vulgaris* L.) infested with the two-spotted spider mite (*Tetranychus urticae* Koch) as their prey. An air pump-based system [15] was used to collect mites for experiments. All experiments were conducted at 25°C, 50% RH and LD 16:8.

### Age synchronization of adult females

The age of the adult females used in the following experiments was synchronized by collecting egg-laying females from the laboratory colonies, allowing them to lay eggs for 24 h, then removing the females and allowing the eggs to hatch and rearing the young until 2 to 3 days after reaching adulthood.

### Detection of solution uptake

A solution of 10% (w/v) Brilliant Blue FCF dye (BB; Wako Pure Chemical, Osaka, Japan) in water was used to evaluate the uptake of the solutions by mites. The distribution of blue color in the alimentary canal was observed using each tested method.

### Delivery methods

#### Soaking

**Fig 1:**
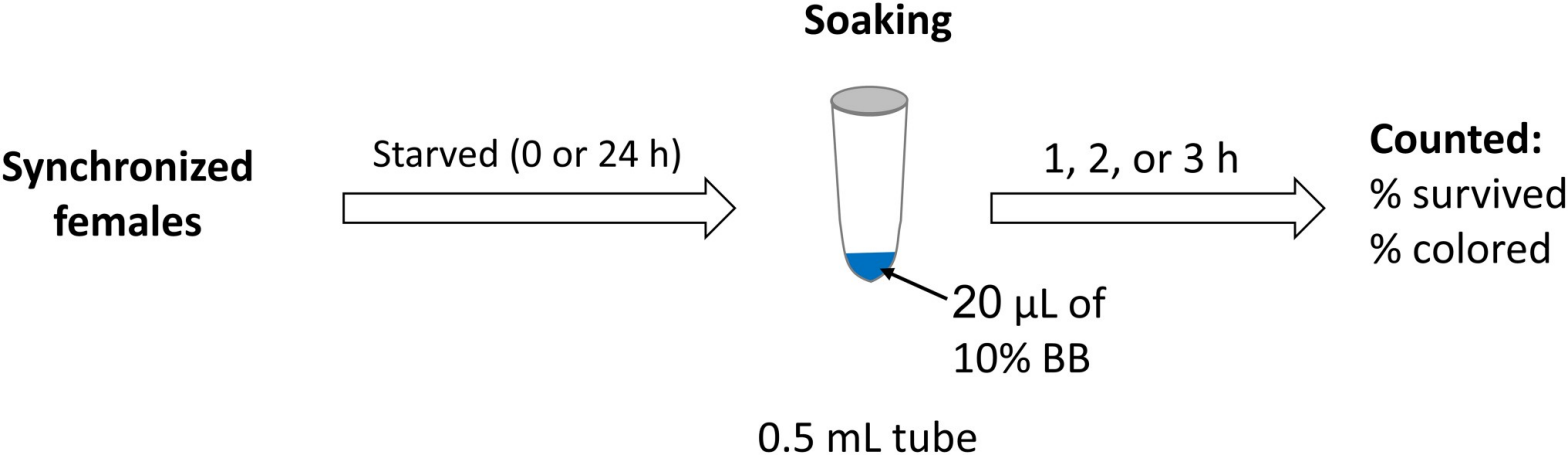
Schematic overview for *N*. *californicus* soaking.

Age-synchronized *N*. *californicus* females were starved in an empty polypropylene tube (1.5 mL) for 24 h. Non-starved females were used as a control. A soaking solution of 10% (w/v) BB in water (20 μL) was prepared in a 0.5-mL polypropylene tube (Fig 1). The starved or non-starved females were released onto a bean leaf disc and a known number of adult females were picked up and immediately transferred to the soaking tubes (20 females per tube/each), where they were allowed to soak for 1, 2, or 3 h. After each soaking trial, all the females were removed from the tubes with a fine brush (Interlon 1026-3/0, Maruzen Artist Materials & Works, Tokyo, Japan). Surviving females were collected from the inner wall of the tube and from the solution, and counted; fatalities were counted also. The number of surviving females in which the alimentary canal was colored with BB was counted. The method was not applied to immature stages of *N*. *californicus* due to the difficulty of soaking such small individuals.

#### Feeding on soaked spider mites

A previously published protocol for soaking adult *T*. *urticae* females [15] was used with a slight modification. Briefly, adult *T*. *urticae* females of random ages were collected in a 0.5-mL tube (20 females per tube) and soaked in a 20 μL solution of 10% (w/v) BB and 0.1% (v/v) Tween 20 in water for 24 h. Simultaneously, age-synchronized females of *N*. *californicus* were starved in a 1.5-mL tube for 24 h. Non-starved females were used as a control. Soaked *T*. *urticae* females were retrieved from the solution and transferred to a new 0.5-mL tube. Starved or non-starved *N*. *californicus* females were transferred to the 0.5-mL tube and allowed to feed on the soaked *T*. *urticae* females for 24 h (~ 3 soaked TSSM adults/ 1 predator individual). The same procedure was applied for *N*. *californicus* protonymphs and deutonymphs, but without the starvation step. For both the adult and immature stages, the numbers of survivors and fatalities were counted after 24 h of feeding on the soaked *T*. *urticae*. The number of surviving mites in which the alimentary canal was colored with BB was also counted.

#### Droplet feeding

**Fig 2:**
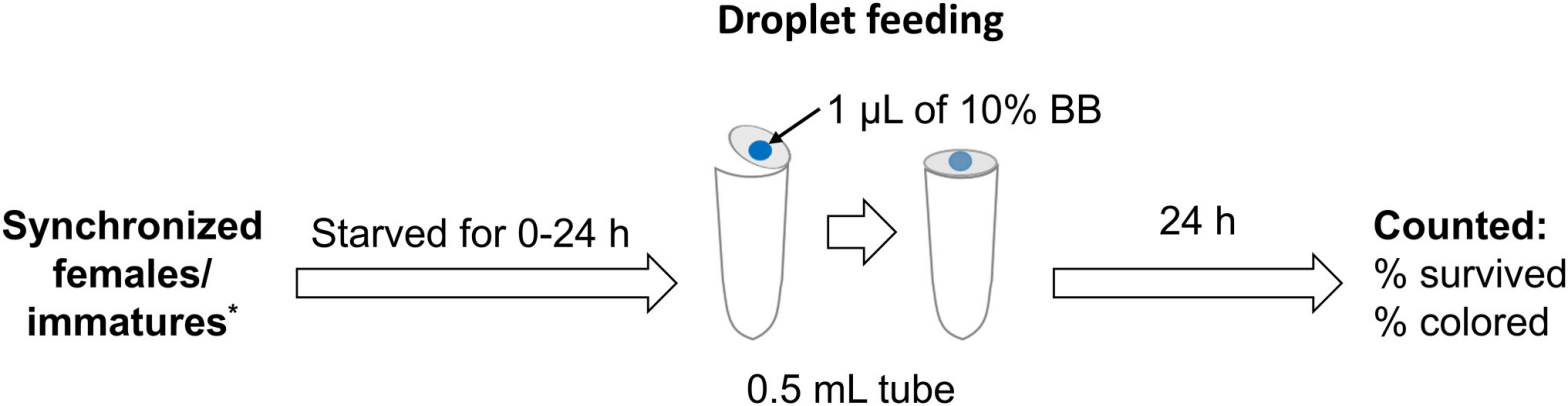
Schematic overview for *N*. *californicus* droplet-feeding. * For immatures droplet-feeding, a starvation period of 24 h was needed to enhance liquid uptake by duetonymphal stage.

Age-synchronized females of *N*. *californicus* were starved in a 1.5-mL tube for 0, 4, 8, 16, or 24 h. After each starvation period, 10 females were transferred to a 0.5-mL tube containing a single droplet (1 μL) of 10% (w/v) BB on the inner surface of the cap; mites were kept in the tube for 24 h (Fig 2). The same procedure was applied for immature stages, but without the starvation step, except deutonymphs, which required a starvation period to increase solution uptake. Due to the short developmental time of the immature stages at 25°C, all larvae and some nymphs reached the subsequent developmental stage within the 24 h of the test. For both adult and immature stages, numbers of survivors and fatalities were counted after 24 h of feeding. The numbers of mites in which the alimentary canal was colored with BB were also counted. The efficiency of the droplet feeding method was farther assessed in a large number of *N*. *californicus* females (*n* = 100 mites per 1 μL droplet in a 0.5-mL tube, 3 replicates). Because the droplet method was most effective for adult *N*. *californicus* (see the Results), we tested this method for use with the specialist predatory mite *P*. *persimilis* using 3-day-old adult females starved for 24 h and then allowed to feed on a droplet for 24 h (20 females per tube, 3 replicates).

#### Feeding on saturated filter paper

Because only adult but no larval feeding was observed using the droplet method (see the Results), we tested an alternate larval feeding method. Larvae were confined in 0.5-mL tubes containing a piece of filter paper (2 × 10 mm) saturated with 10% (w/v) BB (8 μL) instead of the droplet (ca. 20 larvae per tube). Feeding observations were carried out after 3 h.

### Statistical analysis

Data were analyzed using Fisher’s exact test or a chi-squared test depending on sample size. For multiple comparisons, a significance level (α) of 0.05 was corrected using the Bonferroni correction. Analysis was performed using the ‘fifer’ library [16] in the statistical software R 3.4.0 [17]. Python-based plotting with Matplotlib [18] was used for graphical representations. Data represent mean ± SEM.

## Results

**Fig 3:**
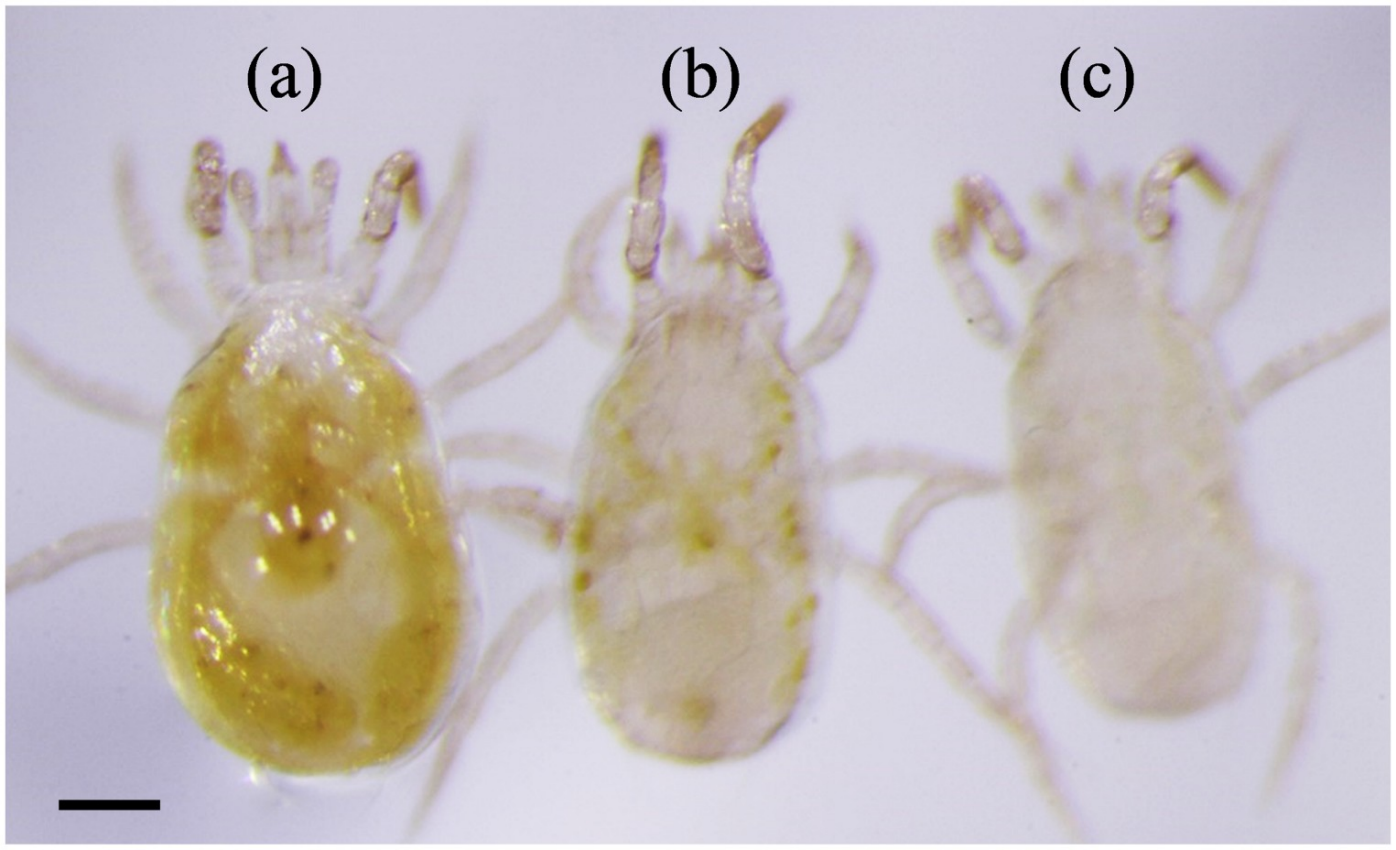
The alimentary canal of *Neoseiulus californicus* adult females without any BB FCF application. (a) non-starved female, (b) female starved for 24 h, and (c) female starved for 24 h followed by 24 h with access to only water. Scale bar: 100 μm.

The alimentary canals of Adult *N*. *californicus* females without any tracer dye application and starved for 0, 24 h or starved for 24 h plus 24 h with only access to water are shown in Fig 3.

### Soaking

**Fig 4:**
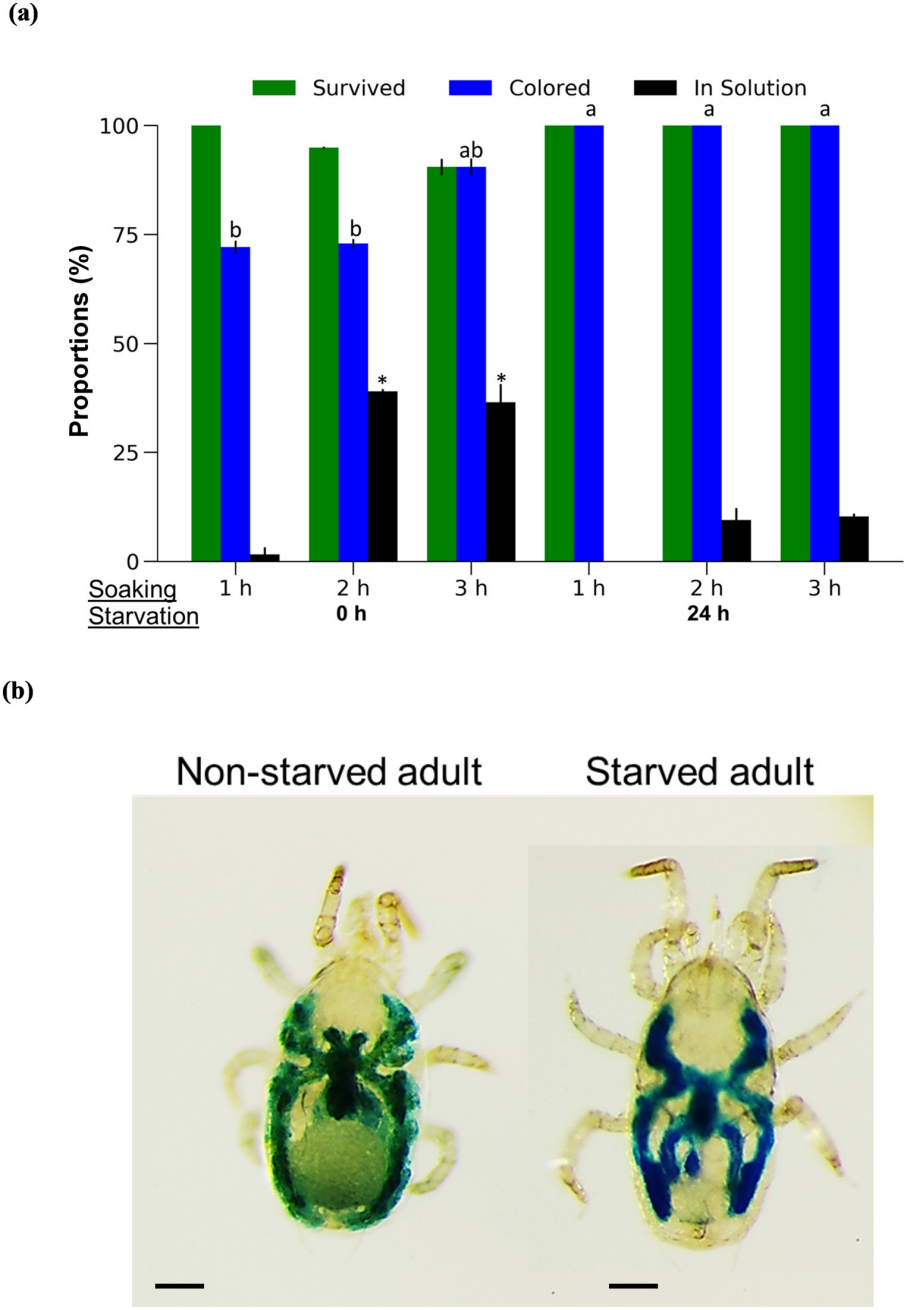
Soaking treatment with and without prior starvation. (a) Proportions of surviving female *Neoseiulus californicus* adults, mites with colored caeca, and mites found in the soaking solution. (b) Blue coloring of non-starved and starved adult females that ingested the solution. Different letters indicate significant differences in the proportion of colored mites. * Significant differences in the proportion of mites found in the soaking solution. *n* = 60 individuals per treatment (20 individuals per replicate, 3 replicates). Chi-squared test with Bonferroni correction. Scale bar: 100 μm.

Adult *N*. *californicus* females soaked for 2 or 3 h without starvation showed 4% and 10% mortality, respectively, whereas soaking duration had no significant effect on survival of mites starved for 24 h (Fig 4a). The alimentary canal of all starved mites was colored by BB ingested from the soaking solution (Fig 4b) and the proportion was significantly higher than that of non-starved mites soaked for 1 or 2 h (χ^2^ = 60.012, *df* = 5, *P* < 0.0001). In non-starved mites, the color of the alimentary canal was greenish, due to mixing of the dye with food material remaining in the alimentary canal. The number of mites collected from the soaking solution was highest with no prior starvation and a soaking duration > 1 h (χ^2^ = 67.495, *df* = 5, *P* < 0.0001).

### Feeding on soaked spider mites

**Fig 5:**
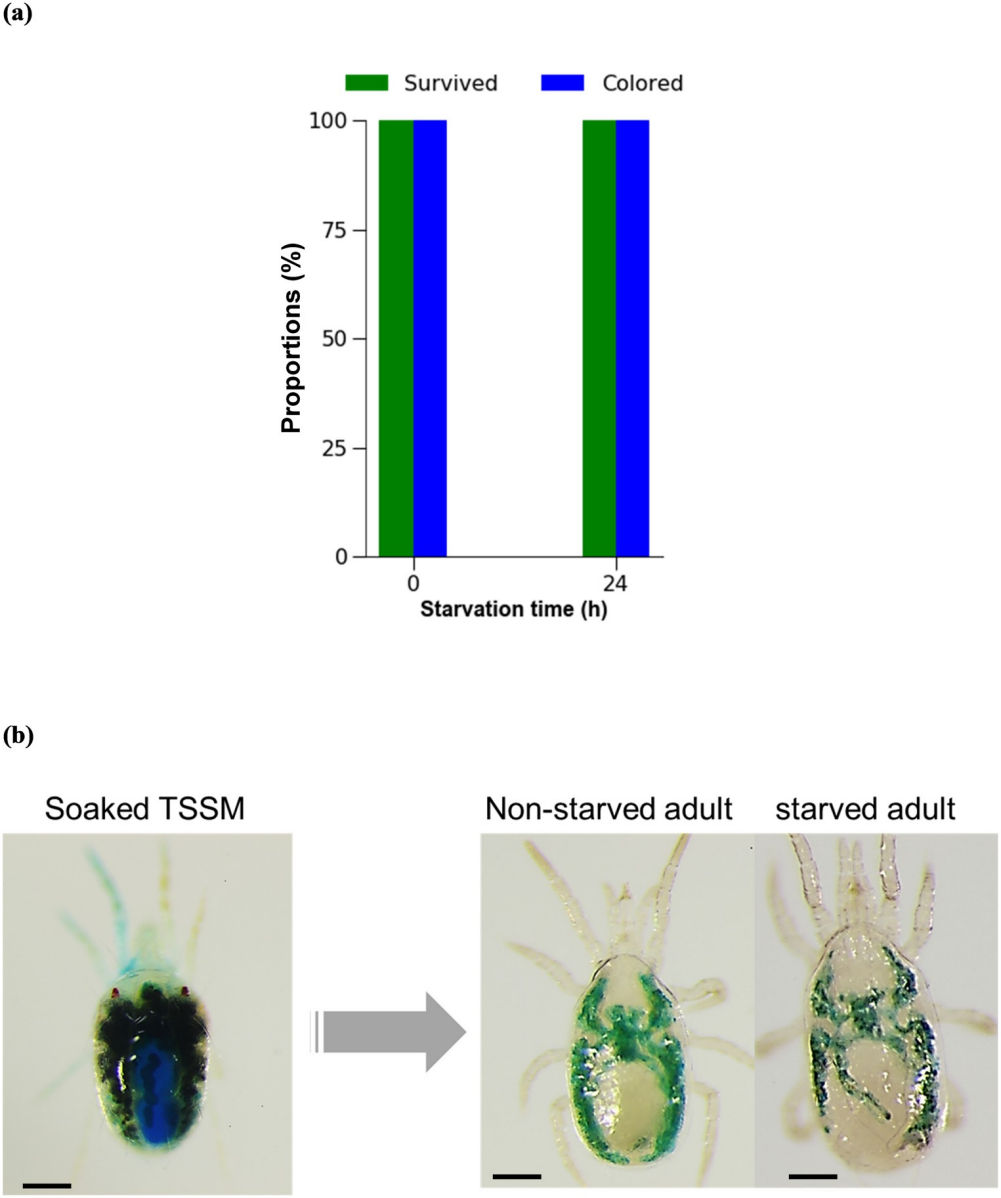
Feeding *Neoseiulus californicus* adults on soaked *Tetranychus urticae* (TSSM). (a) Proportions of surviving and colored female *N*. *californicus* adults after feeding on soaked *T*. *urticae* for 24 h. (b) Blue coloring of soaked *T*. *urticae*, and of starved and non-starved adults fed the soaked *T*. *urticae*. *n* = 60 individuals per treatment (20 individuals per replicate, 3 replicates). *P* > 0.05, Fisher’s exact test. Scale bar: 100 μm.

**Fig 6:**
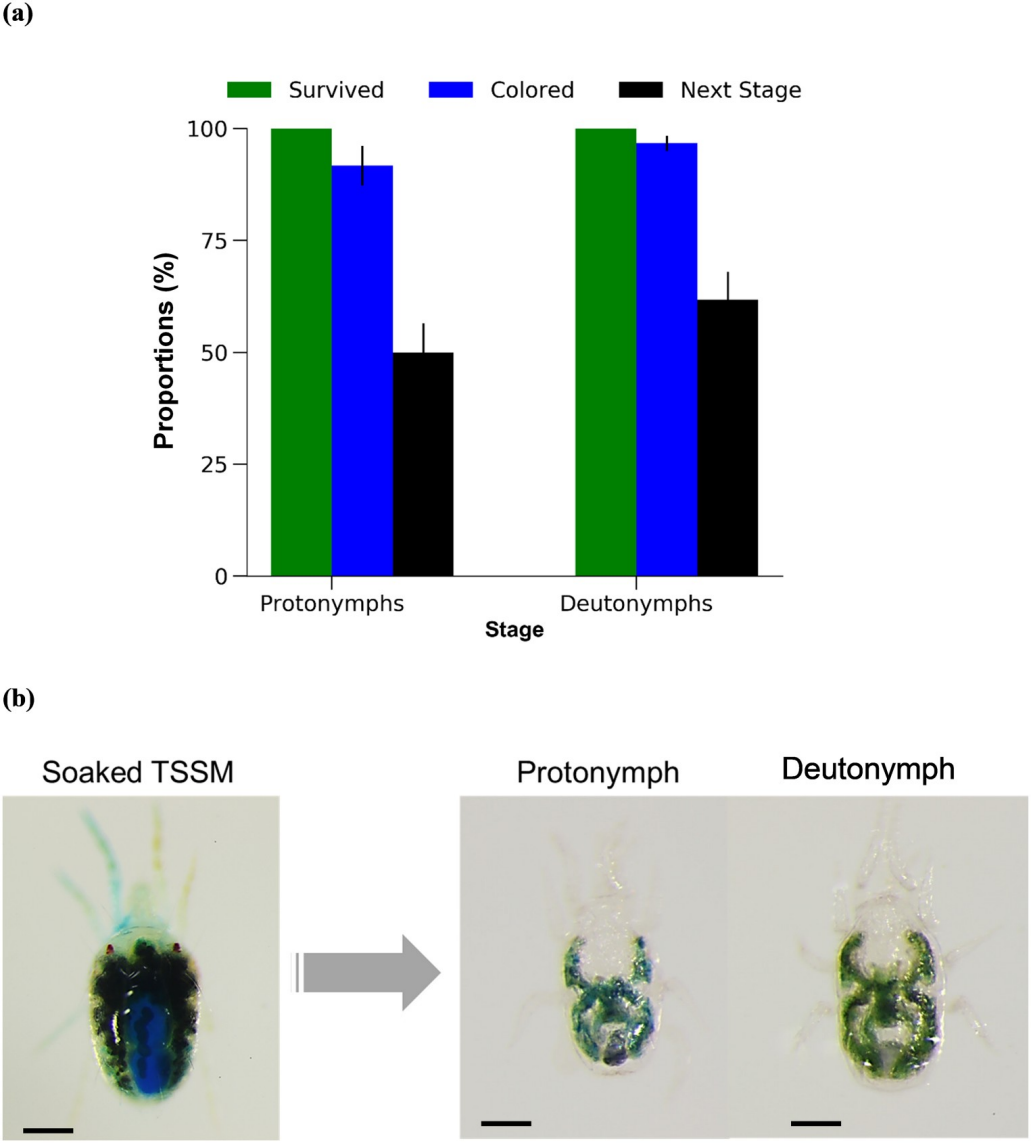
Feeding *Neoseiulus californicus* immature stages on soaked spider mites. (a) Proportions of surviving and colored protonymphs and deutonymphs of *N*. *californicus*, and proportion that molted to the next developmental stage. (b) Blue coloring of soaked *Tetranychus urticae*, and of protonymphs and deutonymphs after feeding on soaked prey mites. No prior starvation. *n* = 60 individuals per treatment (20 individual per replicate, 3 replicates). *P* > 0.05, Fisher’s exact test or chi-squared test with Bonferroni correction. Scale bar: 100 μm.

No mortality was observed in adult females of *N*. *californicus* after 24 h feeding on soaked adult females of *T*. *urticae*, regardless of prior starvation (*P* > 0.05, Fisher’s exact test) (Fig 5a). *Neoseiulus californicus* females with an alimentary canal colored by ingesting tracer dye are shown in Fig 5b. Under both treatments, *N*. *californicus* females laid several eggs during the 24 h of feeding on soaked *T*. *urticae*. Similarly, *N*. *californicus* protonymphs and deutonymphs showed 100% survival and 92% and 97% coloration of the alimentary canal, respectively, after 24 h feeding on soaked *T*. *urticae* (Fig 6a and 6b). In addition, 50–60% of immature individuals molted to the next developmental stage (i.e., protonymphs became deutonymphs and deutonymphs became adults) after 24 h feeding on soaked *T*. *urticae* (Fig 6a).

### Droplet feeding

**Fig 7:**
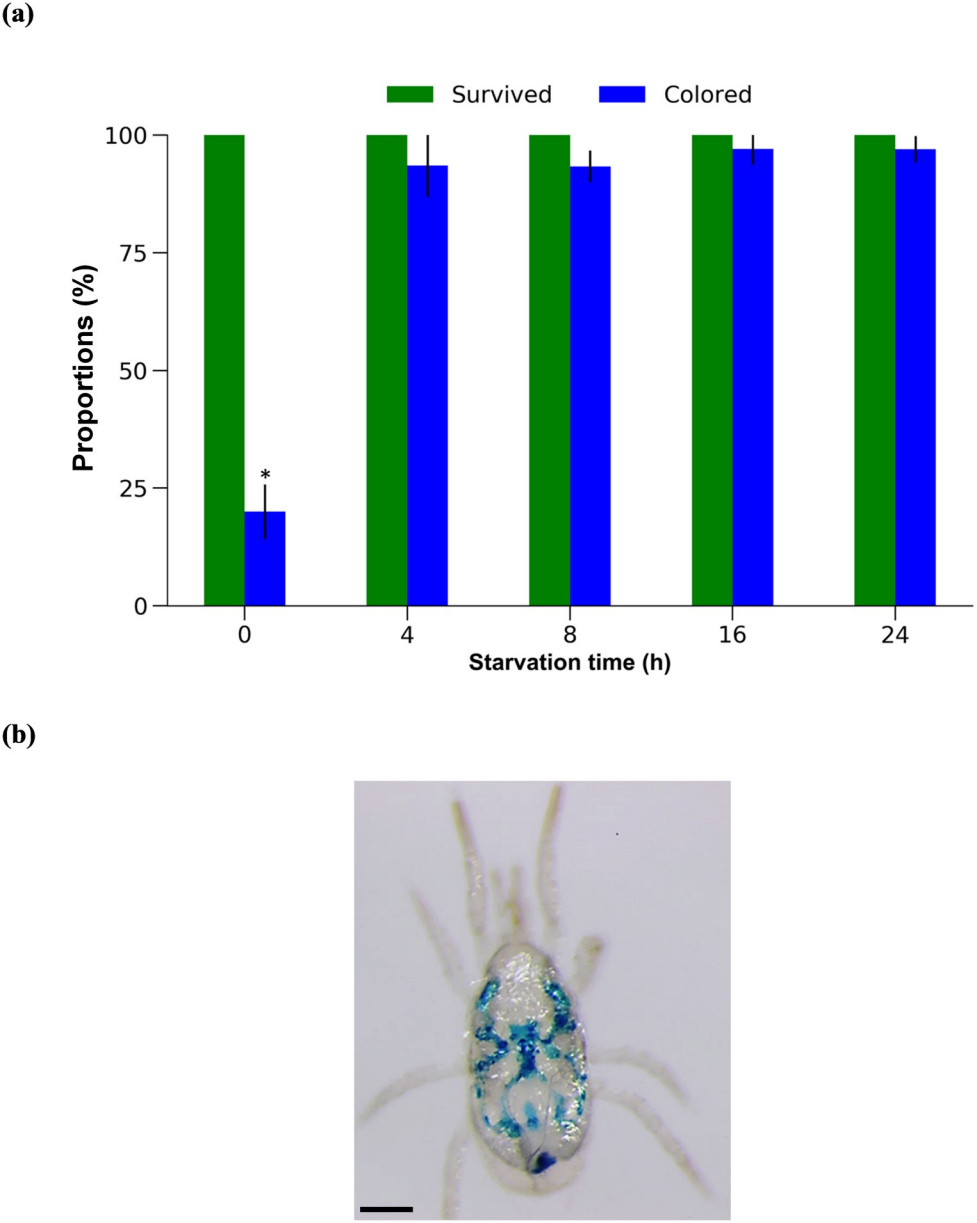
Droplet-fed female *Neoseiulus californicus* adults. (a) Proportions of surviving and colored mites after starvation for 0, 4, 8, 16, or 24 h. (b) Blue coloring of adult female. *n* = 30 mites per treatment (10 mites per replicate, 3 replicates). *P < 0.005, Fisher’s exact test with Bonferroni correction. Scale bar: 100 μm.

No mortality was observed in adult females of *N*. *californicus* after 24 h feeding on droplets on the inner surface of the tube cap, regardless of prior starvation (0 to 24 h) (Fig 7a). However, only 20% of non-starved females showed blue coloration of the alimentary canal (Fig 7b), compared to 93–97% of females starved for 4 to 24 h (*P* < 0.0001, Fisher’s exact test). The supplemental video (S1 Video) shows adult *N*. *californicus* females feeding on the droplet.

In an experiment assessing the efficiency of oral delivery in a larger number of adult females with the droplet feeding method, a 24 h starved adult females (100 mites per replicate, 3 replicates) were confined for 24 h in a tube containing a single droplet (1 μL) of 10% (w/v) BB on the inner surface of the cap. After 24 h of feeding, 93 ± 0.6% of females showed blue coloration of the alimentary canal (S1 Fig). The supplemental video (S2 Video) shows a 100 adult *N*. *californicus* females inside a 0.5-mL tube 1 h after the start of droplet-feeding experiment where the blue alimentary canal is visible in the majority of the females.

**Fig 8:**
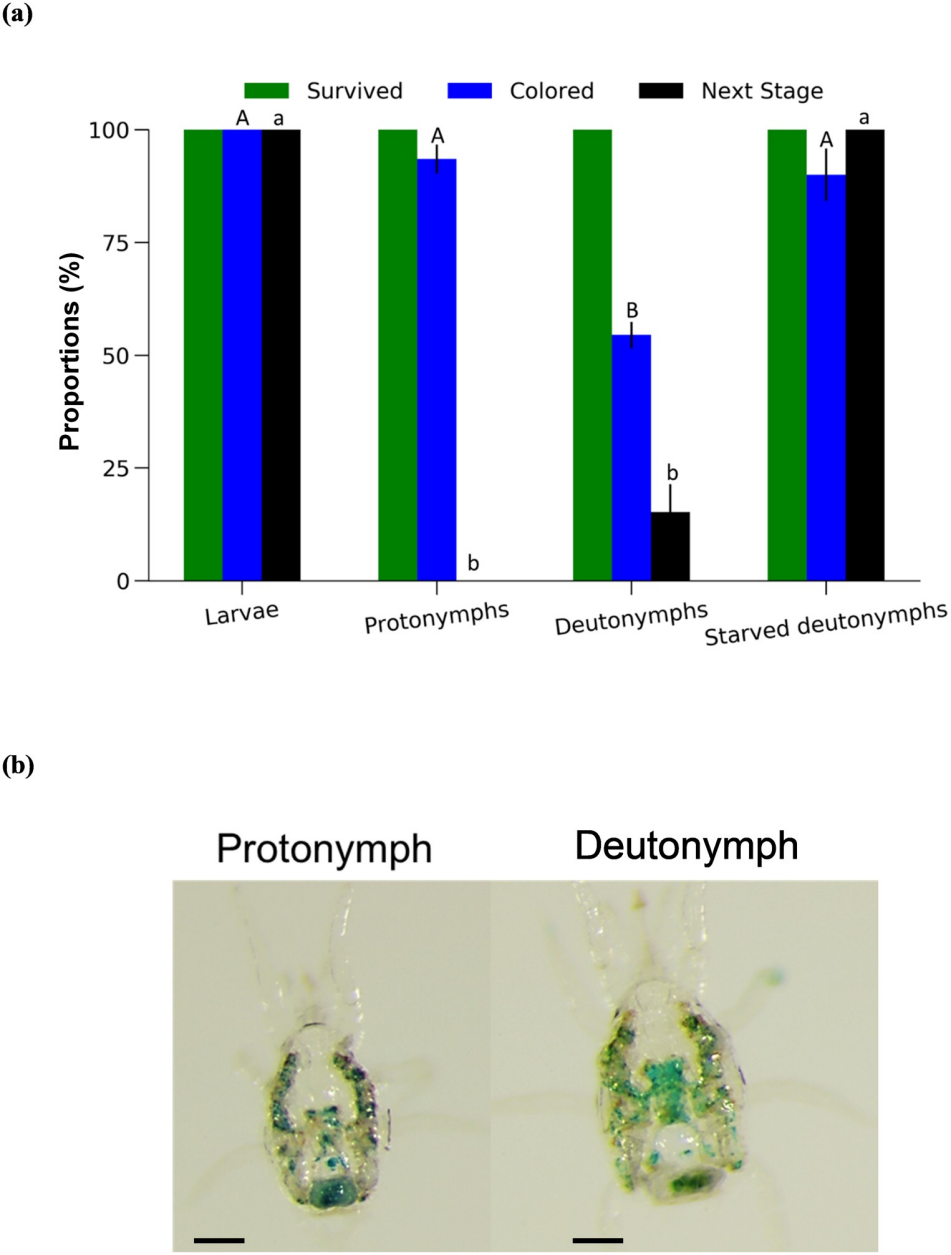
Droplet-fed immature stages of *Neoseiulus californicus*. (a) Proportions of surviving and colored mites, and (b) blue coloring of a protonymph and a deutonymph. *n* = 30 mites per treatment (10 mites per replicate, 3 replicates). Different capital letters indicate significant differences in the proportion of colored mites. Different lowercase letters indicate significant differences in the proportion of mites molted to the following developmental life stage. *P* < 0.05, Fisher’s exact test with Bonferroni correction. Scale bar: 100 μm.

No mortality was observed in immature mites fed on droplets for 24 h (Fig 8a). All larvae reached the protonymphal stage and 100% of emerged protonymphs showed blue coloration of the alimentary canal after 24 h of droplet feeding (Fig 8b). No blue coloration was observed in larvae even when the droplet feeding duration was 6 h, and no larvae reached the protonymphal stage within this time. Nearly 94% of protonymphs showed blue coloration of the alimentary canal (Fig 8b) but no individuals reached the deutonymphal stage under this condition. About half of deutonymphs showed blue coloration of the alimentary canal (Fig 8b) and approximately 15% of deutonymphs reached the adult stage after 24 h of droplet feeding. When deutonymphs were starved for 24 h prior to feeding, all reached the adult stage and 90% showed blue coloration of the alimentary canal after 24 h of droplet feeding.

**Fig 9:**
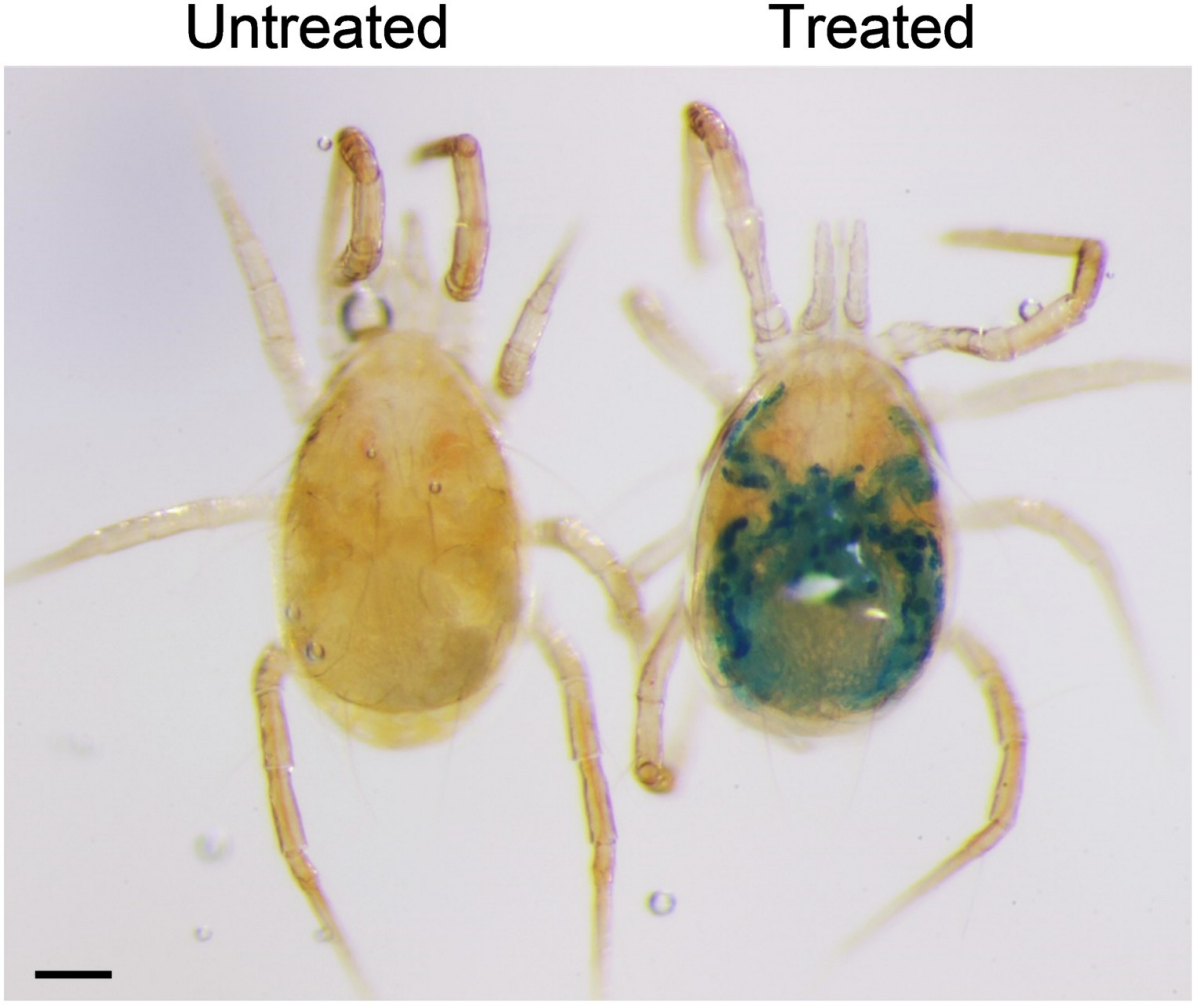
Droplet-fed female *Phytoseiulus persimilis* adults. Adult female of *P*. *persimilis* after feeding on solution dyed with blue tracer dye (brilliant blue FCF) using the droplet-fed method. Blue color is visible in the adult female gastric caeca. Scale bar: 100 μm.

Because droplet feeding was most efficient for oral delivery of BB and required least handling, we also tested this method with the specialist predatory mite, *P*. *persimilis*. An average of 81 ± 4.4% of mites (20 individuals per replicate, 3 replicates) showed blue coloration of the alimentary canal after 24 h of droplet feeding (Fig 9).

### Feeding on saturated filter paper

**Fig 10:**
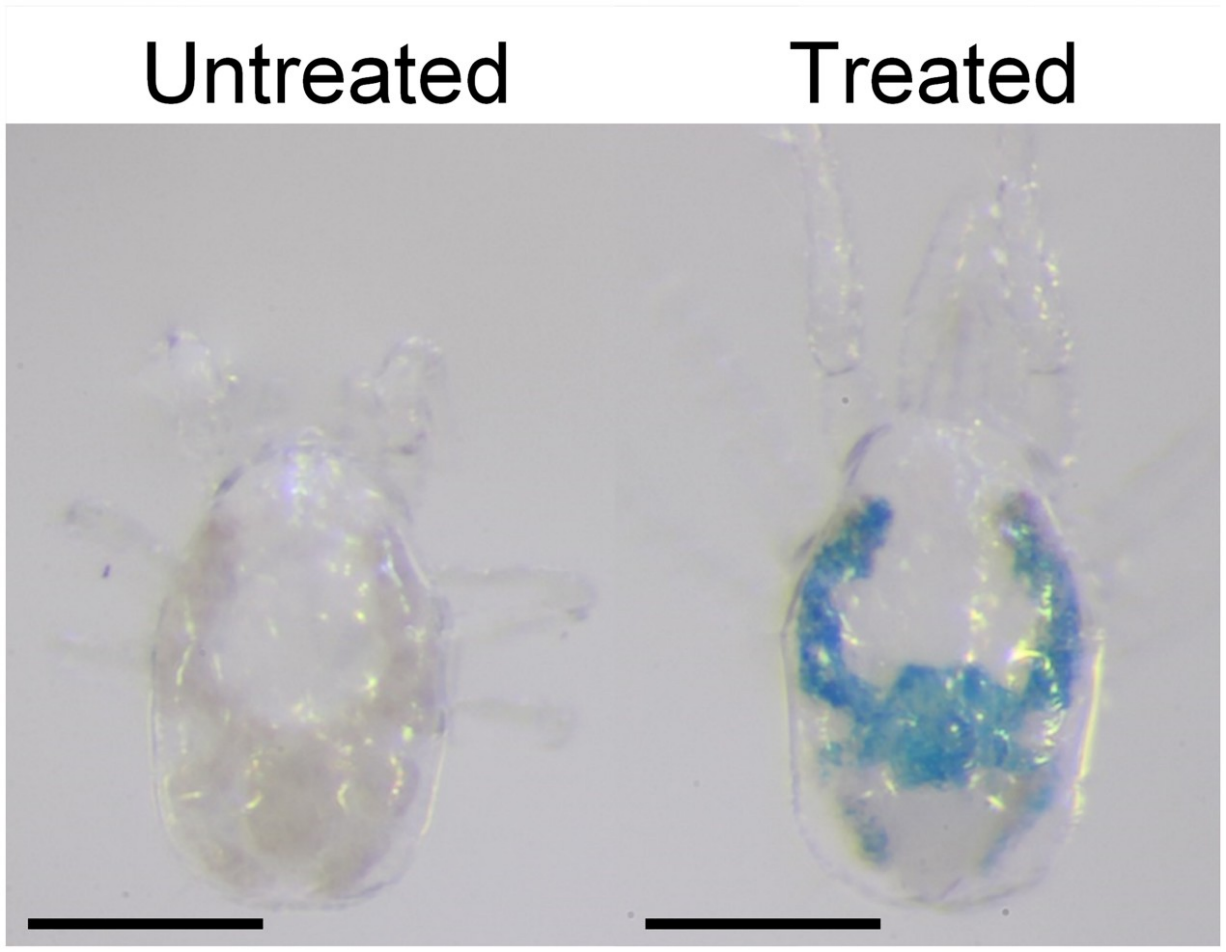
Feeding larva on solution saturated filter paper. Larva of *Neoseiulus californicus* after feeding on filter paper saturated with 10% (w/v) blue tracer dye (brilliant blue FCF). Scale bar: 100 μm.

Although no larvae fed from BB droplets, an average of 71.7 ± 6.0% of larvae showed blue coloration of the alimentary canal after confinement with 10% (w/v) BB-infiltrated filter paper for 3 h (20 individuals per replicate, 3 replicates) (Fig 10).

A general overview of the methods tested for delivering liquid compounds to *N*. *californicus* in the current study is presented in Table 1.

**Table 1:** An overview of the methods tested for delivering liquid compounds to *Neoseiulus californicus*.

Delivery method	Stage	Vol. μL/ individual	Treatment time [h]	Handling	% Colored mites	% Mortality
Soaking	adults	1	1–3	difficult	un-starved: 70–90 starved: 100	4–10 in un-starved mites
Feeding soaked *T*. *urticae*	adults	3 ^a^	24	difficult	100	0
	nymphs				92–97	
Droplet-feeding	adults	0.01–0.1	24	easy	un-starved: 20 Starved: 93–07	0
	deutonymphs				un-starved: 50 starved: 90	
	protonymphs				94	
	larvae				all molted to protonymphs with 100% coloration	
Feeding on solution saturated filter paper	larvae	0.4 ^b^	3	moderate ^c^	72	0

^a^ 1 μL is required for *T*. *urticae* female but ~3 soaked female will be needed to feed *N*. *californicus* for ca. 24 h.

^b^ It may be possible to increase the numbers of larvae.

^c^ According to our result, this is the only way to deliver a liquid solution to larvae. It is also possible to use the same method for adults and nymphs delivery but we found that the droplet method is easier to conduct.

## Discussion

Delivery of liquid substances to phytoseiid mites is relevant for bioassays for pesticide sensitivity, RNAi-based reverse genetics, and commercial production. In this study, we examined the efficiency of four methods for delivery of aqueous solutions to different life stages of the predatory mite *N*. *californicus*. After we determined the most efficient method, we also tested its applicability to the specialist predatory mite *P*. *persimilis*. With all methods tested, an aqueous solution of 10% (w/v) BB was delivered to the caeca of *N*. *californicus*, and the proportion of mites with colored caeca increased when they were starved prior to the feeding experiments.

Feeding by droplet was most efficient, since it only required a small volume of test solution (100 mites/μL), handling required for experimental set-up was simple, and a high proportion of mites showed colored alimentary canals (Fig 7, Table 1). In previous studies, dsRNA was orally delivered to the phytoseiid mite *M*. *occidentalis* by releasing mites starved for 24 h onto a Parafilm disc (22 mm in diameter) containing a 10-μL droplet of solution [9]. Although Wu and Hoy [9] did not report the proportion of mites to which the solution was successfully delivered, they reported that ~10% of mites escaped and some solution evaporated shortly after the beginning of the experiment. Ozawa et al. [10] reported that dsRNA was orally delivered to unreported numbers of *P*. *persimilis* by mites fed via a piece of cotton (1 cm in diameter) saturated with 500 μL of solution placed in a plastic cup (10 cm in diameter). Although the above-mentioned methods were successfully applied for oral delivery of dsRNA and subsequent RNAi-mediated silencing of targeted genes, the droplet feeding method developed in the current study has the potential to be used in high-throughput RNAi screening because of its high efficiency; only 1 μL of test solution is needed for oral delivery to 100 mites in a 0.5-mL closed system that prevents mite escape and evaporation of solution.

Although the droplet feeding method is effective for delivery of a liquid solution to nymphal stages of *N*. *californicus*, it could not be used for larvae. Larvae of *N*. *californicus* are facultative feeders, meaning that they can molt into protonymphs without prior feeding [19]. This may also indicate a lower dispersal tendency in larvae compared to other developmental stages, and the chance of larvae finding the droplet is consequently lower. For this reason, a solution-saturated filter paper was used instead and successfully enabled delivery to larvae.

Some mortality (≈10%) was observed in non-starved *N*. *californicus* after soaking for ≥2 h, and the proportions of colored mites were approximately 70% and 90% when soaked for 1–2 and 3 h, respectively (Fig 4). Mites that were starved prior to soaking showed no mortality or reduction in the proportion of colored mites. We suppose that non-starved mites are heavier than starved ones, making it difficult for the former to exit the soaking solution; the starvation period seemed to have an advantage for delivery of the soaking solution.

Feeding of *N*. *californicus* on soaked *T*. *urticae* was effective for solution delivery, even without prior starvation, and mites showed a caecum colored a dense blue after this treatment (Figs 5 and 6). However, both the predator soaking and feeding on soaked *T*. *urticae* methods have drawbacks: handling of mites of either species is difficult, particularly during soaking, and both methods require a large volume of test solution.

We tested four methods for oral delivery of a liquid substance to the predatory mite *N*. *californicus*. Feeding on a solution droplet was most efficient, owing to the simple protocol and small volume of solution required (1 μL of solution for 100 mites), compared to predator soaking or feeding on soaked *T*. *urticae*. This method was also effective for delivery to the specialist *P*. *persimilis* (Fig 9), indicating that it may be widely applicable to a variety of phytoseiid species. Feeding on solution droplets could be useful for bioassays for RNAi-based reverse genetics and pesticides in predatory mites and may be improved or modified to facilitate the oral delivery of liquid artificial diets for their mass production.

## Supporting information

S1 Video A video shows droplet-fed by female *Neoseiulus californicus* adults. Adult female *N*. *californicus* feeding on a 1-μL droplet of 10% (w/v) blue tracer dye (brilliant blue FCF) on the inner surface of a 0.5-mL polypropylene tube. Normal speed (30 fps).
(MP4)

S2 Video A video shows droplet-fed by 100 females *Neoseiulus californicus* adults. Adult females of *N*. *californicus* feeding on a 1-μL droplet of 10% (w/v) blue tracer dye (brilliant blue FCF) on the inner surface of a 0.5-mL polypropylene tube. The video is taken 1 h after the start of the experiment. The speed is accelerated (2x).
(MP4)

S1 Fig Droplet-fed delivery to a large number of female *Neoseiulus californicus* adults. Adult females (100 mites per replicate, 3 replicates) of *N*. *californicus* after 24 h of feeding on a 1-μL droplet of 10% (w/v) blue tracer dye (brilliant blue FCF). Scale bar: 100 μm.
(DOCX)

## References

[1] van Lenteren JC. The state of commercial augmentative biological control: plenty of natural enemies, but a frustrating lack of uptake. Biocontrol. 2012; 57: 1–20. 10.1007/s10526-011-9395-1

[2] McMurtry JA, Croft BA. Life-styles of Phytoseiid mites and their roles in biological control. Annu Rev Entomol. 1997; 42: 291–321. 10.1146/annurev.ento.42.1.291 15012316

[3] Rhodes EM, Liburd OE. Evaluation of predatory mites and acramite for control of twospotted spider mites in strawberries in north central Florida. J Econ Entomol. 2006; 99: 1291–1298. 10.1603/0022-0493-99.4.1291 16937684

[4] Fraulo AB, Liburd OE. Biological control of twospotted spider mite, *Tetranychus urticae*, with predatory mite, *Neoseiulus californicus*, in strawberry. Exp Appl Acarol. 2007; 43: 109–119. 10.1007/s10493-007-9109-7 17924197

[5] Gerson U, Weintraub PG. Mites for the control of pests in protected cultivation. Pest Manag Sci. 2007; 63: 658–667. 10.1002/ps.1380 17533640

[6] Kola VSR, Renuka P, Madhav MS, Mangrauthia SK. Key enzymes and proteins of crop insects as candidate for RNAi based gene silencing. Front Physiol. 2015; 6: 119 10.3389/fphys.2015.00119 25954206 PMC4406143

[7] Yu N, Christiaens O, Liu J, Niu J, Cappelle K, et al Delivery of dsRNA for RNAi in insects: an overview and future directions. Insect Sci. 2013; 20: 4–14. 10.1111/j.1744-7917.2012.01534.x 23955821

[8] Sun D, Guo Z, Liu Y, Zhang Y. Progress and prospects of CRISPR/Cas systems in insects and other arthropods. Front Physiol. 2017; 8: 608 10.3389/fphys.2017.00608 28932198 PMC5592444

[9] Wu K, Hoy MA. Oral delivery of double-stranded RNA induces prolonged and systemic gene knockdown in *Metaseiulus occidentalis* only after feeding on *Tetranychus urticae*. Exp Appl Acarol. 2014; 63: 171–187. 10.1007/s10493-014-9772-4 24509787

[10] Ozawa R, Nishimura O, Yazawa S, Muroi A, Takabayashi J, et al Temperature‐dependent, behavioural, and transcriptional variability of a tritrophic interaction consisting of bean, herbivorous mite, and predator. Mol Ecol. 2012; 21: 5624–5635. 10.1111/mec.12052 23043221

[11] McMurtry JA, de Moraes GJ, Sourassou NF. Revision of the lifestyles of phytoseiid mites (Acari: Phytoseiidae) and implications for biological control strategies. Syst Appl Acarol. 2013; 18: 297–320. 10.11158/saa.18.4.1

[12] Castagnoli M, Simoni S. *Neoseiulus californicus* (McGregor) (Acari: Phytoseiidae): survey of biological and behavioral traits of a versatile predator. Redia. 2003; 86: 153–164.

[13] Hamlen RA, Lindquist RK. Comparison of two *Phytoseiulus* species as predators of twospotted spider mites on greenhouse ornamentals. Environ Entomol. 1981; 10: 524–527. 10.1093/ee/10.4.524

[14] Sabelis MW. Life history. Capacity for population increase 1985 In: Helle W, Sabelis MW, editors. Spider mites, their biology, natural enemies and control, vol 1B. Amsterdam: Elsevier pp. 35–41.

[15] Suzuki T, España MU, Nunes MA, Zhurov V, Dermauw W, et al Protocols for the delivery of small molecules to the two-spotted spider mite, *Tetranychus urticae*. PLOS ONE. 2017; 12(12): e0190025 10.1371/journal.pone.0190025 29244858 PMC5731762

[16] Fife D. Fifer: a biostatisticians toolbox for various activities, including plotting, data cleanup, and data analysis. R package version 1.1. 2017. https://CRAN.R-project.org/package=fifer

[17] R Core Team. R: A language and environment for statistical computing. Vienna, Austria; 2017 https://www.R-project.org/

[18] Hunter JD. Matplotlib: A 2D Graphics Environment. Comput Sci Eng. 2007; 9: 90–95. 10.1109/MCSE.2007.55

[19] Chittenden AR, Saito Y. Why are there feeding and nonfeeding larvae in phytoseiid mites (Acari, Phytoseiidae)? J Ethol. 2001; 19: 55–62. 10.1007/s101640170018

