# Supplementary figures and images for: Oral delivery of water-soluble compounds to the phytoseiid mite Neoseiulus californicus (Acari: Phytoseiidae)
Source: PLoS One. 2019 Oct 16;14(10):e0223929. doi: 10.1371/journal.pone.0223929 (PMC6795443; doi:10.1371/journal.pone.0223929)

**S1 Fig**


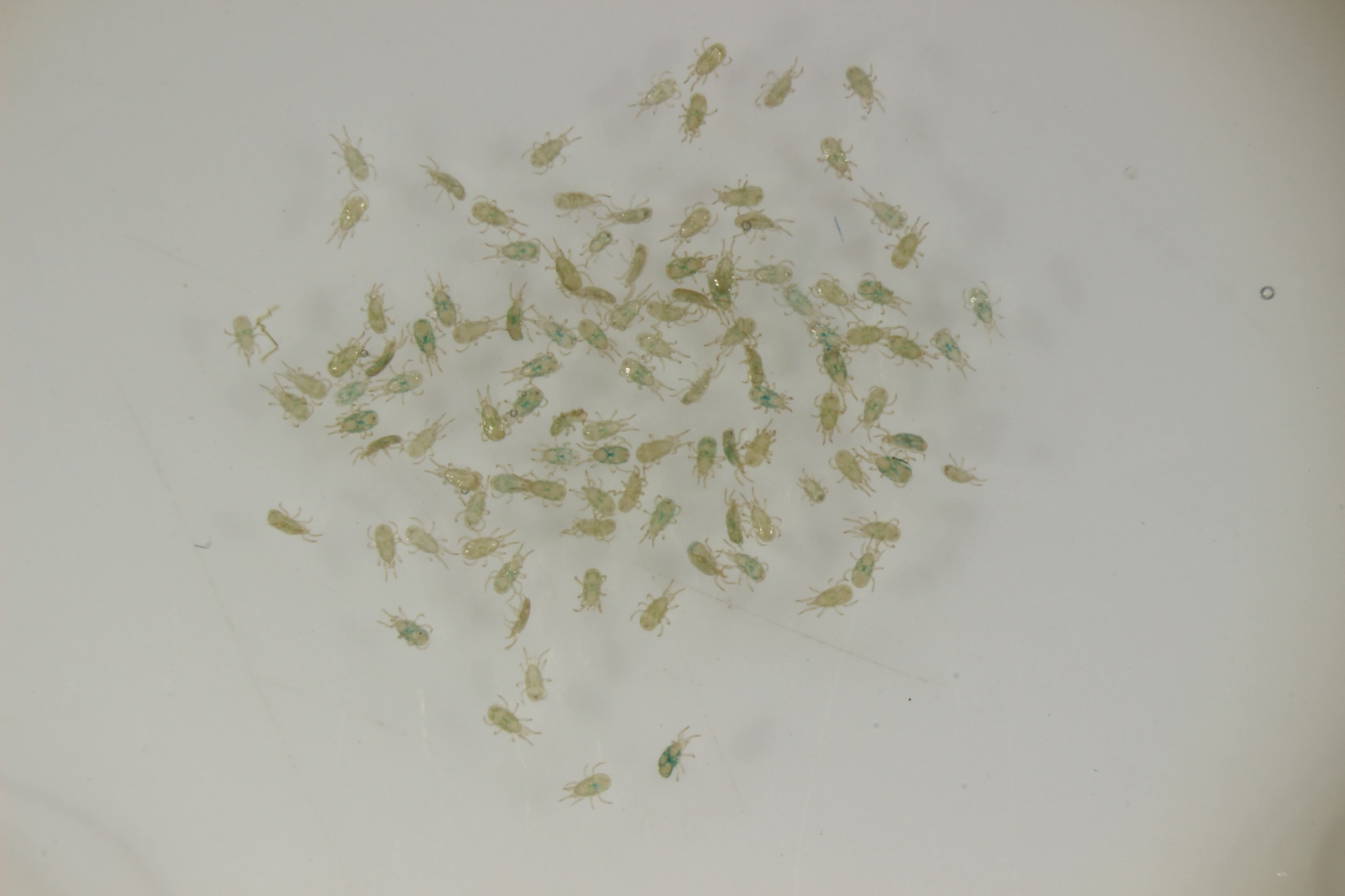

Supplement: S1 Fig — Adult females (100 mites per replicate, 3 replicates) of N. californicus after 24 h of feeding on a 1-μL droplet of 10% (w/v) blue tracer dye (brilliant blue FCF). Scale bar: 100 μm. (DOCX) [file pone.0223929.s003.docx]
